# Titanium oxide nanomaterials as an electron-selective contact in silicon solar cells for photovoltaic devices

**DOI:** 10.1186/s11671-023-03803-x

**Published:** 2023-03-11

**Authors:** Dongkyun Kang, Jongwon Ko, Changhyun Lee, Donghwan Kim, Hyunju Lee, Yoonmook Kang, Hae-Seok Lee

**Affiliations:** 1 Department of Materials Science and Engineering, Korea University, Seoul, 0284 Korea; 2 Meiji Renewable Energy Laboratory, Meiji University, 1-1-1 Higashimita, Tama-Ku, Kawasaki, 214-8571 Japan; 3 KU-KIST Green School, Graduate School of Energy Environment, Korea University, Seoul, Korea

**Keywords:** Titanium oxide nanomaterials, Electron selective contact, Passivation effect

## Abstract

To obtain high conversion efficiency, various carrier-selective contact structures are being applied to the silicon solar cell, and many related studies are being conducted. We conducted research on TiO_2_ to create an electron-selective contact structure that does not require a high-temperature process. Titanium metal was deposited using a thermal evaporator, and an additional oxidation process was conducted to form titanium oxide. The chemical compositions and phases of the titanium dioxide layers were analyzed by X-ray diffraction. The passivation effects of each titanium oxide layer were measured using the quasi-steady-state photoconductance. In this study, the layer properties were analyzed when TiO_2_ had a passivation effect on the silicon surface. The charge and interface defect densities of the layer were analyzed through CV measurements, and the passivation characteristics according to the TiO_2_ phase change were investigated. As a result, by applying optimized TiO_2_ layer thickness and annealing temperature conditions through the experiment for passivation to the cell-like structure, which is the structure before metal and electrode formation, an implied open-circuit voltage (iVoc) of 630 mV and an emitter saturation current density (*J*_0_) value of 60.4 fA/cm^2^ were confirmed.

**Supplementary Information:**

The online version contains supplementary material available at 10.1186/s11671-023-03803-x.

## Introduction

Generally, silicon solar cells form a p–n junction through doping processes to separate photogenerated carriers. However, the carrier-selective contact structure separates carriers by aligning and using the energy band between the carrier selection layer and silicon [1]. Materials such as polysilicon [2], transition metal oxides [3–10], and amorphous silicon [11] have been applied as carrier-selective layers to silicon solar cells. The carrier selectivity of these layers, which affects the efficiency of solar cells, depends on the band structure of the material deposited on the silicon. Among the various materials used as the carrier-selective layer, titanium oxide deposited on the interface of n-type silicon, which has a large valence band offset and, a small conduction band offset is suitable for use as an electron-selective layer [12]. Titanium oxide is a material that has been studied as an anti-reflection coating in silicon solar cells and as an electron-transfer material in organic solar cells [13]. Methods for depositing titanium oxide using atomic layer deposition (ALD) to increase electron selectivity in the n^+^ region have been studied, which have good passivation property [3]. Approximately 20% of high-efficiency cells have been reported when using an ALD titanium oxide layer, and various mass-production attempts using these methods are underway [14].

In this study, we deposited titanium metal using a thermal evaporator and then used a tube furnace to form an oxide layer through annealing. The experiments were conducted in accordance with our previous study [15]. To improve the passivation properties of titanium oxide nanomaterials, a thin tunnel silicon oxide layer was formed at the silicon–TiO_2_ interface. The lifetime results of the samples using the titanium oxide layer through thermal evaporation were compared, and then the passivation property was analyzed to determine the cause of this difference. Through the optical and electrical measurement results, the band structure of the layer acting as electron-selective contacts on the crystalline silicon was confirmed. As a result, the possibility of using a titanium oxide nanomaterial layer as a carrier selection layer was confirmed.

## Materials and methods

### Sample preparation

**Fig. 1:**
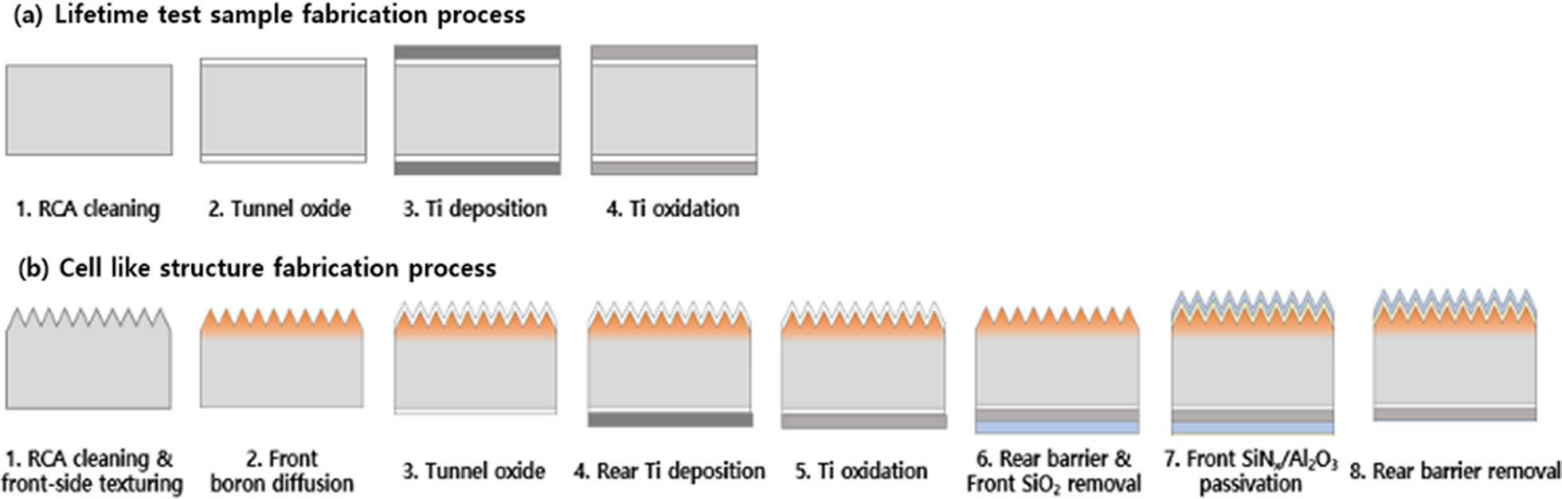
Fabrication process of **a** lifetime and **b** cell-like structure samples

First, as shown in Fig. 1a, double-side symmetric structures were prepared to measure minority carrier lifetime. The n-type float-zone (Fz) crystalline silicon wafers with a thickness of ~ 280 μm and resistivity of ~ 2.5 Ω cm were used. The wafers were cleaned with the cleaning process of the Radio Corporation of America (RCA) using ammonium peroxide mixture (APM), and hydrochloric peroxide mixture (HPM) solutions. Approximately 1.4 nm of the nanoscale silicon dioxide layer was formed on the wafer surface using 30% hydrogen peroxide solution at 80 °C for 10 min. The Ti metal source was evaporated using the thermal evaporation method. After the evaporation step over both sides of the sample, oxidation processes were performed in the tube furnace (250–350 °C).

A cell-like structure, as shown in Fig. 1b, using an electron-selective contact was prepared with an n-type float-zone (FZ) silicon wafer (100) with a thickness of ~ 280 µm and resistivity of ~ 1 Ω∙cm. The wafers were cleaned using the RCA cleaning method, and then a surface texturing process was performed on the front surface. The surface texturing process formed a random pyramid structure using the alkaline-based unisotropic etching methods. The p^+^ emitter was formed through a diffusion process using boron tribromide, and this process was carried out at about 1010 °C using a tube furnace. After this process, borosilicate glass (BSG) is formed on the surface of the emitter, which is removed using dilute hydrofluoric acid, and the sheet resistance range of the emitter was 100–120 Ω/sq. A silicon oxide layer with a thickness of ~ 1.4 nm was grown on the wafer surface via wet chemical oxidation using H_2_O_2_. The thickness and refractive index of the silicon oxide layer were measured using ellipsometer; the results can be seen in Additional file 1: Figure S1. Subsequently, a titanium oxide layer was prepared by using a thermal evaporator with a titanium metal source. In order to change metallic titanium into a titanium oxide layer, an annealing process was performed in various temperature ranges in an oxygen atmosphere in a tube furnace. After depositing an etch barrier on the titanium oxide layer, the thin silicon oxide layer on the front surface was removed, and a front surface passivation layer was deposited. For the passivation layer, an aluminum oxide layer was deposited using ALD, and a SiNx layer was deposited as an anti-reflection coating (ARC) using the PECVD method.

### Sample measurement

WCT-120 (Sinton instruments) was used to confirm the passivation property of the SiO_2_ and TiO_2_ stacked passivation layer, and recombination of the excess carrier concentration (∆*n*) was assumed to occur according to the lifetime equation [16]. The passivation property of samples was compared with the measured effective lifetime (τ_eff_) results of photogenerated carriers and the implied open-circuit voltage (iVoc) of the samples under 1-sun condition with xenon flash lamp was measured with the quasi-steady-state (QSS) mode. iVoc is determined by the following equation [17]:

$${\text{iVoc}} = \frac{kT}{q}\ln \frac{{\left( {N_{{\text{D}}} + \Delta n} \right)\Delta n}}{{n_{{\text{i}}}^{2} }}$$

where *kT* is the thermal energy, *q* is the electric charge, *N*_D_ is the doping concentration, ∆*n* is the excess carrier concentration related to the effective lifetime, and *n*_*i*_ is the intrinsic carrier concentration. The grazing incidence X-ray diffraction (GIXRD) method was used to confirm the phase change of the titanium oxide layer, and the thickness and uniformity of the silicon oxide layer were confirmed using a Stokes laser ellipsometer (Gaertner Scientific Corporation) at nine different points after deposition on the polished wafer. In addition, the CV measurement method was used to measure and confirm the interface defect density and fixed charge values of the titanium oxide layer. The measurement was performed under a − 10 to 15 V voltage by contacting the tip of the mercury probe to the oxide layer.

## Results and discussion

**Fig. 2:**
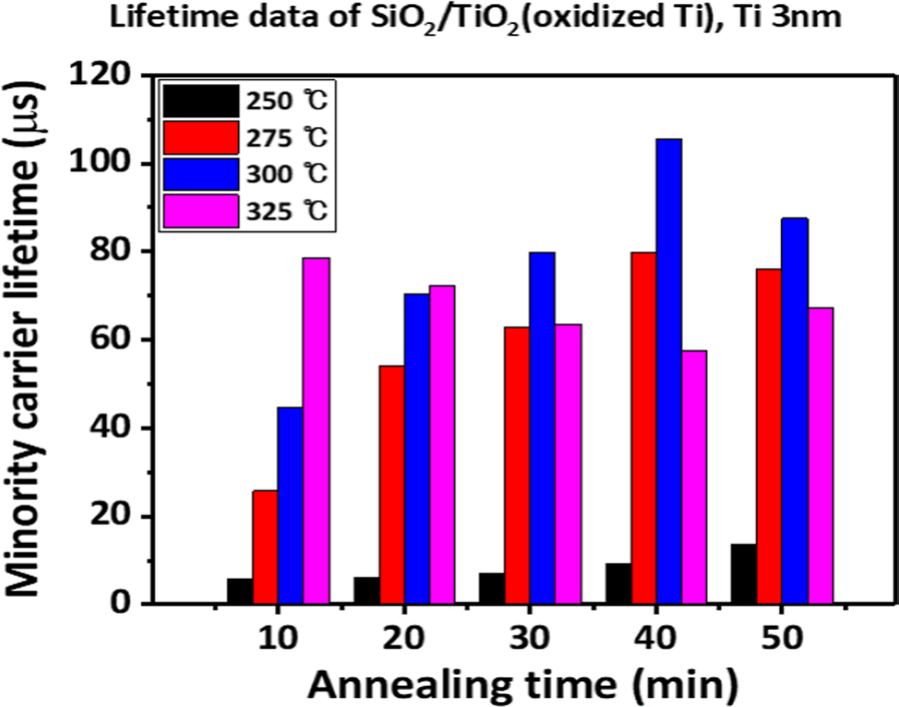
Minority carrier lifetime with a variation in oxidation temperature

**Fig. 3:**
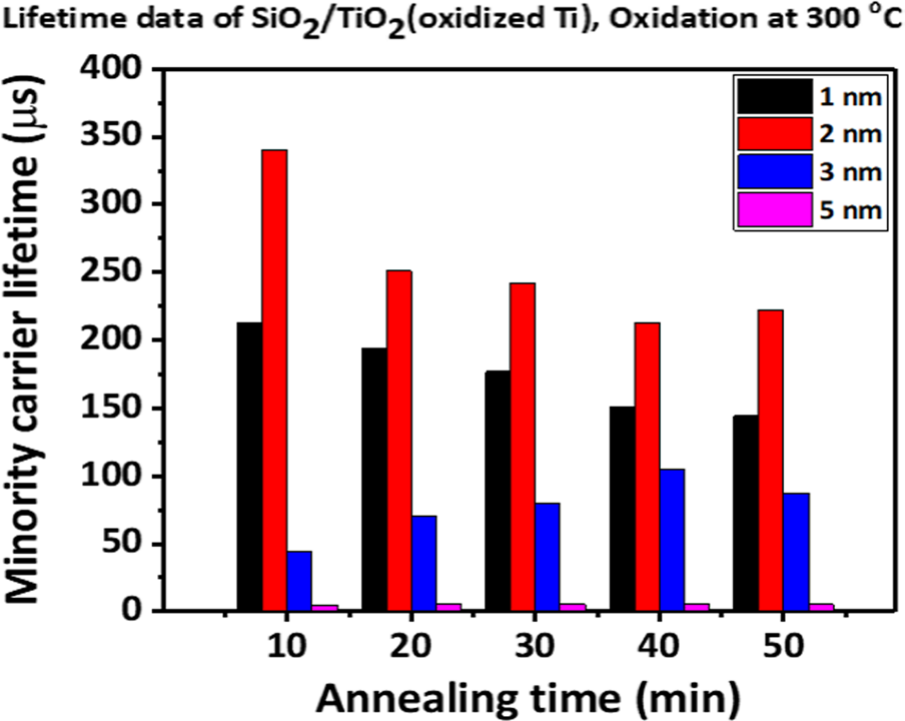
Minority carrier lifetime with a variation in metal thickness

To fabricate a titanium oxide layer, 3-nm-thick metallic titanium deposited using thermal evaporation was carried out in an oxygen atmosphere in a tube furnace by increasing the annealing temperature from 250 to 325 °C with 25 °C increment for 10–50 min. When 3 nm of titanium was annealed at various temperatures, effective carrier lifetimes of ~ 100 μs were obtained for samples annealed 40 min at 300 °C (Fig. 2). Subsequently, the annealing temperature was fixed at 300 °C and the thickness of the titanium metal was changed from 1 to 5 nm. Finally, as shown in Fig. 3, when 2 nm of titanium metal was annealed for 10 min at 300 °C, a lifetime value of approximately 350 μs was obtained. As the thickness of titanium decreases under the same annealing conditions, it is expected that the passivation characteristics will be improved because the oxidation of the Ti metal is better. However, when it becomes smaller from 2 to 1 nm, the lifetime decreases slightly, which is expected to be influenced by the change in the composition of the oxide layer through continuous oxygen atmosphere annealing after conversion [15, 18].

**Fig. 4:**
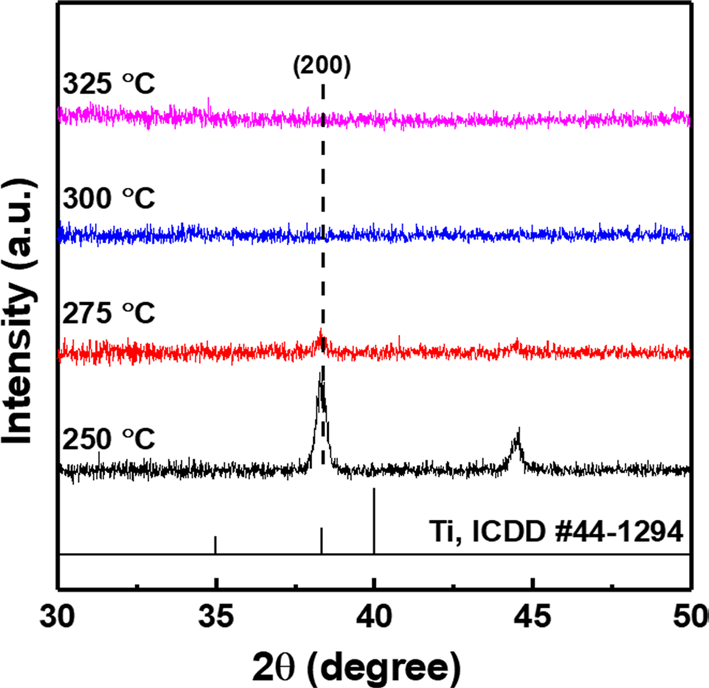
XRD data according to oxidation temperature

**Fig. 5:**
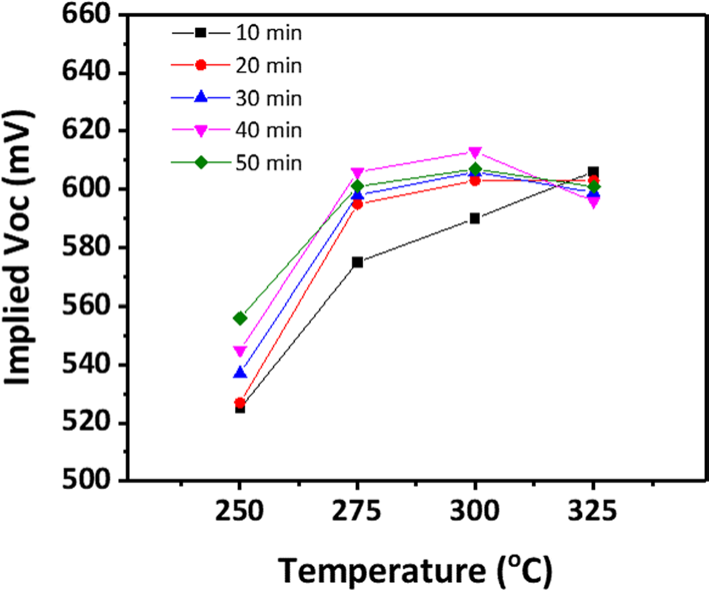
Implied Voc data according to oxidation temperature and time

GIXRD measurements were performed to confirm the phase of the optimized layer. In the case of 300, 325 °C annealing, the titanium oxide was amorphous, but when the annealing temperature was low, metallic peaks appeared on the layer (Fig. 4). This peak was identified as a (200) plane peak corresponding to ICDD #44-1284. A titanium oxide layer with these metallic properties has worse passivation properties than an amorphous layer (Fig. 5). It has been reported that titanium oxide exhibits the best passivation properties when it is in an amorphous phase and deteriorates when a phase change occurs in the layer [19]. This metallic property increases the rate of recombination and the saturation current of carriers on the silicon surface, which degrades the passivation property [15].

**Fig. 6:**
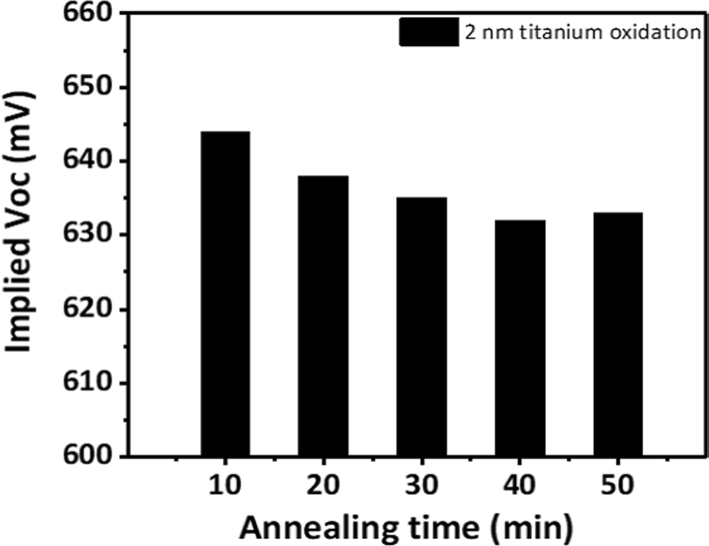
Implied Voc data of 2 nm oxidized titanium oxide according to annealing time

As shown in Fig. 6, after the optimization process, the sample in an amorphous form after annealing at 300 °C exhibited an iVoc value of 644 mV after annealing for 10 min. The carrier lifetime decreased after 20 min of annealing; however, no significant change was observed with the additional annealing process. The optimized layer was confirmed to be in the form of TiO_2_.

**Fig. 7:**
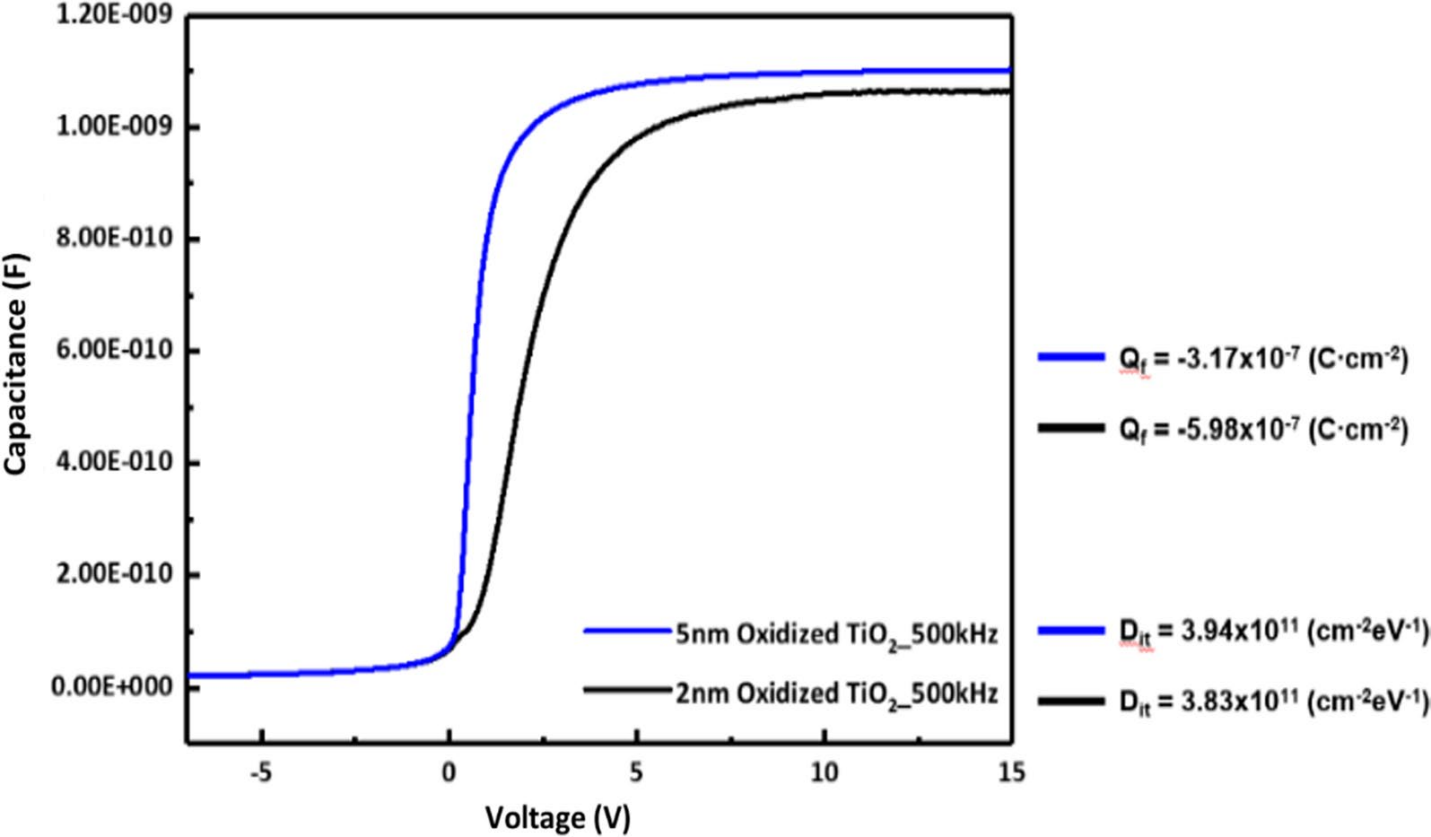
Capacitance–voltage curves of oxidized TiO_2_ according to thickness

**Fig. 8:**
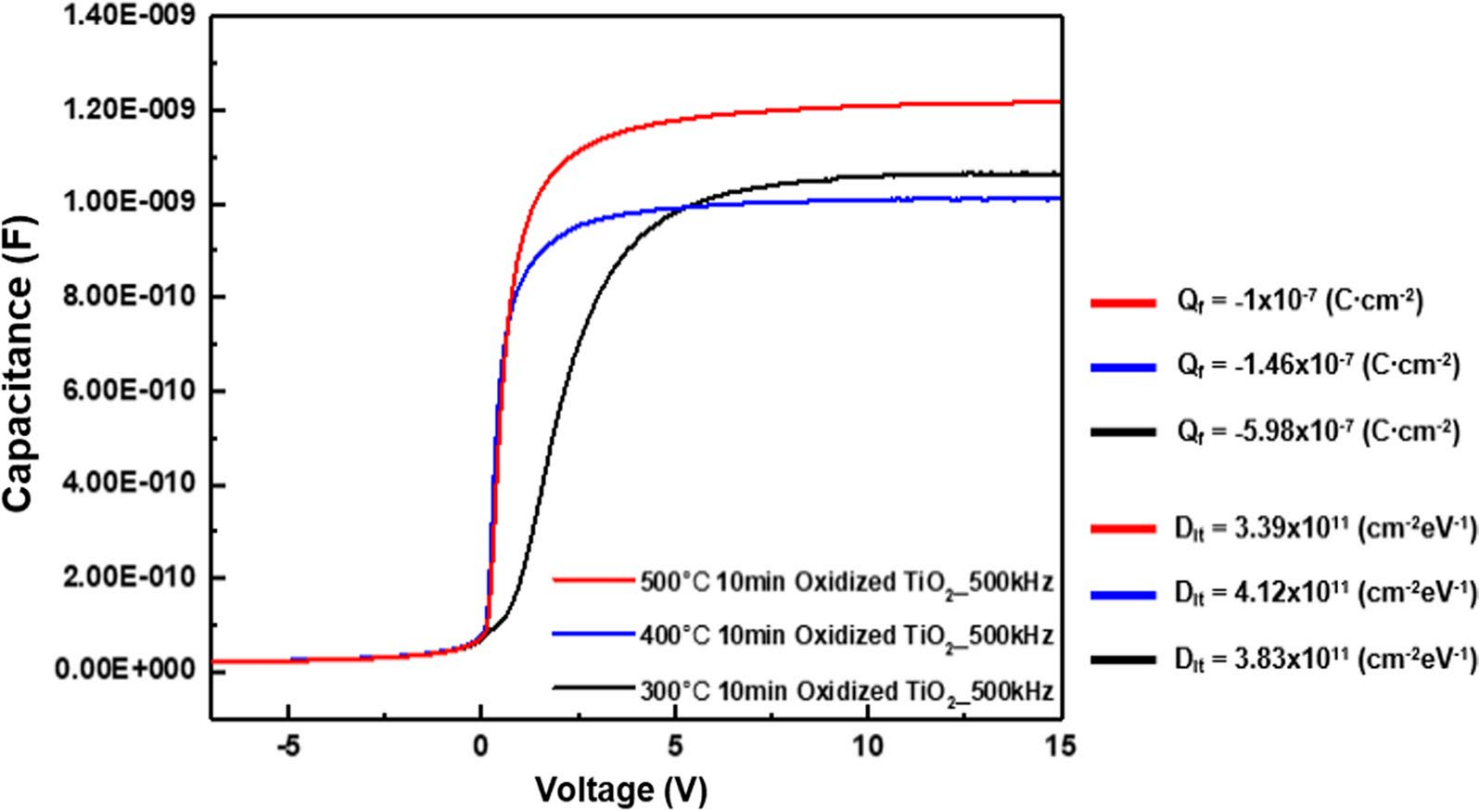
Capacitance–voltage curves of oxidized TiO_2_ according to temperature

Capacitance voltage measurements were performed to identify the cause of the difference in the passivation property of the oxidized titanium oxide layer according to the annealing temperature and time. The fixed charge and interface defect density of the metal oxide can be measured using CV measurements, which are important factors affecting the passivation properties. Figure 7 shows the results of oxidation at 300 °C with various thicknesses of the titanium metal, and Fig. 8 shows the results of oxidation of 2 nm titanium at various temperatures. The results showed that there was no significant difference in the *Q*_f_ and *D*_it_ of the oxidized titanium oxide layer under both conditions. Thus, it can be inferred that the passivation property of the layer is affected by the work function change according to the state of the layer, and not by *Q*_f_ and *D*_it_. The main cause for determining *D*_it_ in this structure is occurred by the chemical passivation of the silicon dioxide layer, and it was confirmed that there is little change in the passivation effect of the silicon dioxide layer under the temperature condition. It is expected to increase the minority carrier lifetime due to the electron selectivity by the work function. The results of work function difference measured by ultraviolet photoelectron spectroscopy (UPS) were reported by Lee et al. [15] and the fabricated structure is expected to have similar results.

**Fig. 9:**
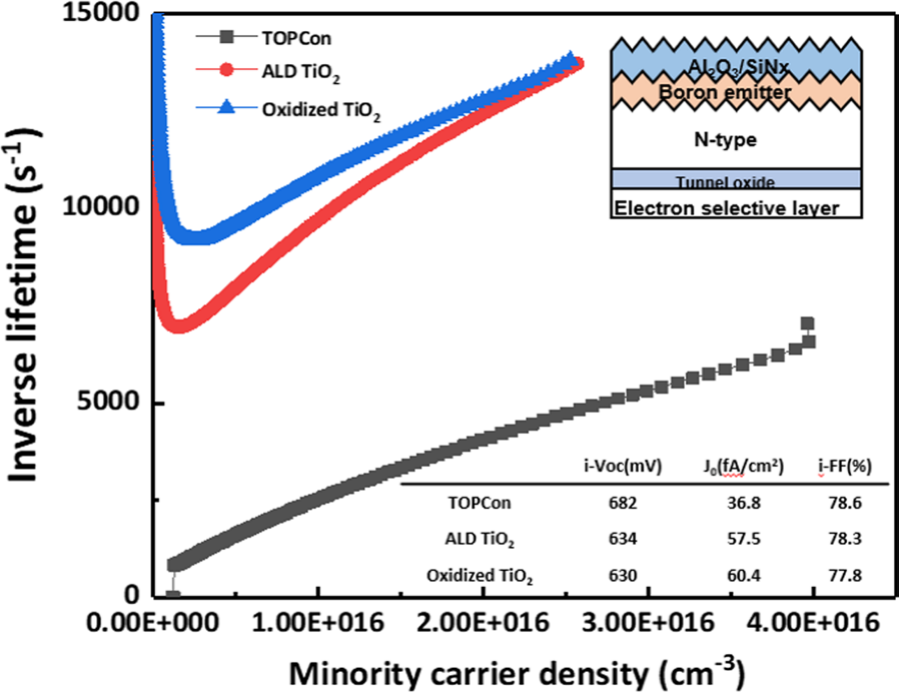
Inverse lifetime data of cell-like structures

Figure 9 shows the cell-like structure of titanium oxide electron-selective contact. With tunnel oxide passivating contact (TOPCon) structure (black dot with line), it showed a *J*_0_ value of 36.8 fA/cm^2^, and when titanium oxide was applied as a rear structure (blue dot with line), a *J*_0_ value of 60.4 fA/cm^2^ was obtained. Also, for the reference data, we optimized TiO_2_ layer by ALD. In the case of titanium oxide deposited by ALD (red dot with line), a *J*_0_ value of 57.5 fA/cm^2^ was obtained. The oxidized TiO_2_ sample is expected to pseudo-efficiency of 19.5%. It is calculated by using measured iVoc and iFF of cell-like structure and simulated *J*_sc_ (39.79 mA/cm^2^) value. The electrical property of TiO_2_ layer is indicated in Additional file 1: Figure S2. It shows the contact resistivity of the TiO_2_ layer at about 0.6 Ω∙cm.

## Conclusion

An electron-selective titanium oxide layer was fabricated using a thermal evaporator, followed by oxygen annealing. The properties of the titanium oxide layer, such as the passivation property, structures, and electrical property, of the layer formed through the above process were investigated. The passivation property was improved by applying thin silicon oxide (~ 1.4 nm), which is capable of tunneling carriers, and optimizing the Si/SiO_2_/TiO_2_ multilayer. For the symmetric structure sample (TiO_2_/SiO_2_/*n-*Si/SiO_2_/TiO_2_), ~ 644 mV of iVoc was obtained after 2 nm of Ti deposition and annealed 10 min at 300 °C. GIXRD measurements showed that when the oxidation process was carried out at a temperature below 300 °C, metal peaks were obtained from inside the layer, indicating the poor passivation property of the layer. XRD measurements confirmed that TiO_2_ was formed without metallic properties when titanium was oxidized at temperatures above 300 °C. The passivation property of the titanium oxide differed depending on the phase of the layer and the annealing time. And, when the thickness of the initially deposited titanium was too thick, a low minority carrier lifetime characteristic was confirmed. CV measurements confirmed that the *Q*_f_ and *D*_it_ of the TiO_2_ layer did not show significant differences depending on the annealing conditions. A difference in passivation property is expected because of the work function variation that occurs as the phase changes according to the annealing conditions, and this requires additional analysis. The pseudo-efficiency of cell-like structure using oxidized TiO_2_ layer confirmed 19.5%. Further research is needed to improve the passivation quality of the layer, and it is necessary to develop a metallization process to manufacture a solar cell to which this layer is applied.

## Supplementary Information

**Additional file 1.**

## Data Availability

The data that support the findings of this study are available from the corresponding author upon reasonable request.
