# Supplementary material for: Titanium oxide nanomaterials as an electron-selective contact in silicon solar cells for photovoltaic devices
Source: Discov Nano. 2023 Mar 11;18(1):39. doi: 10.1186/s11671-023-03803-x (PMC10214925; doi:10.1186/s11671-023-03803-x)
Supplement: Supplementary file 1 — Additional file 1. [file 11671_2023_3803_MOESM1_ESM.docx]

Titanium oxide nanomaterials as an electron selective contact in silicon solar cells for photovoltaic devices

Dongkyun Kang ^1^, Jongwon Ko ^1^, Changhyun Lee ^1^, Donghwan Kim ^1^, Hyunju Lee ^2^, Yoonmook Kang ^3,^*, and Hae-Seok Lee ^3,^*

^1^Department of Materials Science and Engineering, Korea University, Seoul 0284

^2^Meiji Renewable Energy Laboratory, Meiji University, 1-1-1 Higashimita, Tama-ku, Kawasaki 214-8571, Japan

^3^KU-KIST Green School, Graduate School of Energy Environment, Korea University

*Corresponding Authors e-mail: ddang@korea.ac.kr (Y.K.), lhseok@korea.ac.kr (H.-S.L.)


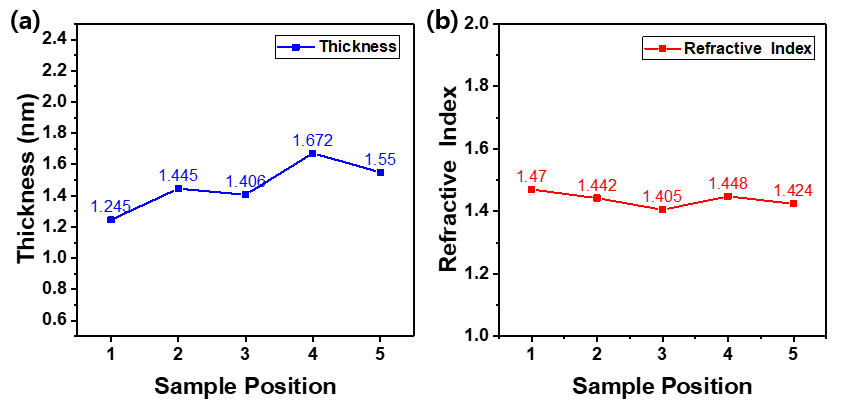


Figure S1. Thickness and Refractive Index of silicon dioxide layer formed by wet chemical oxidation


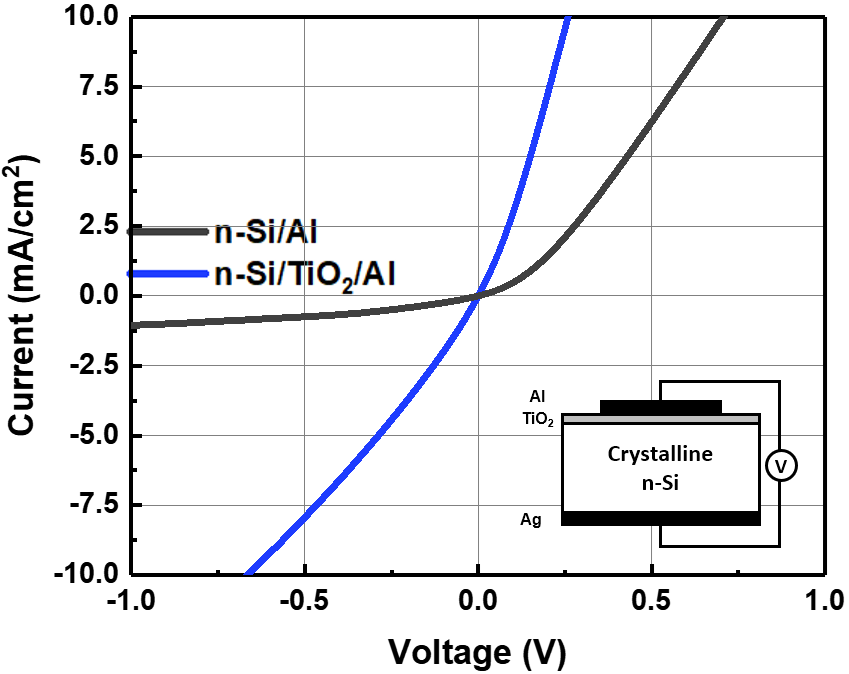


**Figure S2. DIV curves and contact resistivity of TiO_2_ contact**
